# Prediction of Abdominal Aortic Aneurysm Growth Using Geometric Assessment of Computerized Tomography Images Acquired During the Aneurysm Surveillance Period

**DOI:** 10.1097/SLA.0000000000004711

**Published:** 2020-12-29

**Authors:** Anirudh Chandrashekar, Ashok Handa, Pierfrancesco Lapolla, Natesh Shivakumar, Elisha Ngetich, Vicente Grau, Regent Lee

**Affiliations:** * Nuffield Department of Surgical Sciences, University of Oxford, Oxford, United Kingdom; † Department of Engineering Science, University, of Oxford, Oxford, United Kingdom

**Keywords:** abdominal aortic aneurysms, curvature, geometric modeling, undulation

## Abstract

We investigated the utility of geometric features for future AAA growth prediction.

**Background:**

Novel methods for growth prediction of AAA are recognized as a research priority. Geometric feature have been used to predict cerebral aneurysm rupture, but not examined as predictor of AAA growth.

**Methods:**

Computerized tomography (CT) scans from patients with infra-renal AAAs were analyzed. Aortic volumes were segmented using an automated pipeline to extract AAA diameter (APD), undulation index (UI), and radius of curvature (RC). Using a prospectively recruited cohort, we first examined the relation between these geometric measurements to patients' demographic features (n = 102). A separate 192 AAA patients with serial CT scans during AAA surveillance were identified from an ongoing clinical database. Multinomial logistic and multiple linear regression models were trained and optimized to predict future AAA growth in these patients.

**Results:**

There was no correlation between the geometric measurements and patients' demographic features. APD (Spearman *r* = 0.25, *P <* 0.05), UI (Spearman *r* = 0.38, *P <* 0.001) and RC (Spearman *r* =–0.53, *P <* 0.001) significantly correlated with annual AAA growth. Using APD, UI, and RC as 3 input variables, the area under receiver operating characteristics curve for predicting slow growth (<2.5 mm/yr) or fast growth (>5 mm/yr) at 12 months are 0.80 and 0.79, respectively. The prediction or growth rate is within 2 mm error in 87% of cases.

**Conclusions:**

Geometric features of an AAA can predict its future growth. This method can be applied to routine clinical CT scans acquired from patients during their AAA surveillance pathway.

Abdominal aortic aneurysms (AAA) are an abnormal degenerative condition characterized by pathological dilatations of the abdominal region of the aorta. Clinically, an AAA is defined when the aortic diameter is >50% of the healthy aorta adjacent to the aneurysm. The natural history of an untreated AAA consists of progressive dilatation with eventual rupture and death. The clinical management of AAAs consist of screening/diagnosis, regular surveillance, and timely surgical intervention by open surgical repair or endovascular stent grafting.^1,2^

Methods for the prediction of AAA growth is considered as a priority for research in the opinions of vascular and endovascular surgeons.^3^ Accurate prediction of AAA growth in patients can allow for the optimization of surveillance intervals and better inform the timing for surgery. Our prior work has highlighted the feasibility of AAA growth prediction using physiological and biochemical measurements obtained from patients.^4–6^ These measurements, however, require additional research steps to the routine clinical care pathway.

Computerized tomography (CT) scans are utilized extensively as diagnostic tests in medicine and surgery. Globally around 150 million CT scans are performed each year.^7,8^ In the management pathway of AAAs, each patient requires 1 dedicated CT scan before surgery to plan for the operative approach. During the small AAA surveillance period, a proportion of these patients would have also undertaken CT scan(s) for other medical reasons. In these patients, these historic CT scan(s) performed sometime before AAA surgery could serve as the “baseline” scan. The subsequent pre-operative CT scan would serve as a serial scan and enables growth rate calculation during the time period between the 2. With relevant regulatory approval, these historic baseline CT scans can be anonymized and utilized to discover novel features to predict future AAA growth.

As AAAs enlarge, a variety of geometrical changes are observed including altered aortic tortuosity^9^ and increased aneurysmal asymmetry.^10^ Several of these changes result in a unique nonuniform distribution of wall stress and have been hypothesized to either favor AAA growth deceleration or increase rupture risk.^11,12^ Undulation is a measure of the degree of surface irregularity and asymmetry. Undulation index (UI) of cerebral aneurysms has been utilized to quantify its risk of rupture.^13,14^ In this regard, regions of increased curvature [radius of curvature (RC)] within the aneurysm segment contributes to non-laminar fluid flow, and non-uniform wall shear stress.^15,16^ No prior literature has examined the utility of UI or RC for AAA growth prediction. Here we hypothesized that aneurysms with an increased degree of surface undulation and with local regions of increased curvature are prone to rapid growth.

## Methods

### Patient Cohort

The study was conducted as part of the ongoing Oxford Abdominal Aortic Aneurysm (OxAAA) study (Ethics approval Ref: SC/0250/13). The study complies with the principles outlined in the Declaration of Helsinki. Details regarding the OxAAA study cohort and recruitment process have been published.^17^ This study cohort consists of 2 arms.

In the first arm, participants were prospectively recruited to the OxAAA study at the time of surgery. Demographic information were recorded from each patient which were matched to the preop-erative CT scan. Height and weight of the patient were measured to calculate their body mass index. The history of coronary artery disease is defined by angina, myocardial infarction, and/or previous coronary interventions (angioplasty or bypass). The history of peripheral arterial occlusive disease is defined by intermittent claudication, critical limb ischemia and/or previous lower limb arterial intervention (angioplasty or bypass). The history of cerebral arterial disease is defined by transient ischaemic attack or stroke. The history of hypertension, diabetes mellitus, and hypercholesterolemia were as diagnosed by the primary care/general practitioner. We further measured the individual’s blood pressure, blood cholesterol profile, and HbA1c level to ascertain the effect of their pharmacological therapy. Their current medications were further recorded (Table 1).

**Table 1:** Summary of Participant Demographics at the Pre-surgical Assessment and Significance of Spearman Correlation With Extracted Geometric Parameters

		Significance of Spearman Correlation Coefficient		
	All Participants (n = 102)	Diam.	UI	RC
Male (%)	99 (97)	0.35	0.36	0.09
Age at consent (median/IQR)	72 (67–79)	0.12	0.83	0.70
Height (±SD)	1.75 ± 0.08	0.07	0.45	0.06
Weight (Median/IQR)	81.9 (74–90.2)	0.17	0.15	0.07
BMI (Median/IQR)	26.8 (24.3–28.7)	0.61	0.35	0.18
MAP (±SD)	102.2 ± 12.8	0.13	0.70	0.20
Current smoker (%)	24 (24)	0.57	0.99	0.09
Past smoking Hx (%)	68 (67)	0.97	0.48	0.59
Never smoked (%)	13 (13)	0.65	0.34	0.44
CAD Hx (%)	33 (32)	0.25	0.15	0.56
Coronary intervention (%)	26 (25)	0.63	0.41	0.88
PAOD History (%)	16 (16)	0.85	0.59	0.22
Cerebral art. disease (%)	12 (12)	0.10	0.16	0.34
HTN history (%)	74 (73)	0.53	0.77	1.00
Hypercholesterolemia (%)	61 (60)	0.61	0.67	0.75
Tot. cholesterol (median/IQR)	3.8 (3.2–4.6)	0.23	0.76	0.63
HDL (median/IQR)	1.1 (0.9–1.3)	0.47	0.41	0.06
LDL (median/IQR)	1.5 (0–2.5)	0.26	0.92	0.37
TG (median/IQR)	1.2 (0.8–1.6)	0.49	0.65	0.66
Diabetes (%)	16 (16)	0.88	0.26	0.78
HbA1C (median/IQR)	5.6 (5.4–5.9)	0.19	0.81	0.41
Diabetes - oral/insulin (%)	12 (12)	0.19	0.22	0.17
CKD - eGFR < 60 (%)	28 (27)	0.15	1.00	0.96
Creatinine (median/IQR)	86.5 (73.3–101.3)	0.19	0.07	0.74
Beta-blockers (%)	32 (31)	0.68	0.30	0.49
ACEI/ARB (%)	56 (55)	0.27	0.77	0.97
Aspirin (%)	47 (46)	0.83	0.89	1.00
Thienopyridine (%)	9 (9)	0.55	0.17	0.44
Ticragrelor (%)	3 (3)	0.81	0.64	0.72
Anticoagulant (%)	12 (12)	0.93	0.46	0.21
CCBs (%)	43 (42)	0.13	0.64	0.34
Diuretics (%)	22 (22)	0.47	0.38	0.58
Gastro-restraint (%)	31 (30)	0.57	0.68	0.32
Steroids (%)	7 (7)	0.36	0.97	0.15
Statins (%)	74 (73)	0.57	0.76	0.24
AAA Diam (Median/IQR)	63 (58–72.5)			
UI ( SD)	0.23 0.08			
RC (Median/IQR)	35.9 (29.7–46.9)			

Participant demographics were collected at the pre-surgical assessment and were correlated against the extracted geometric parameters of AAA diameter, undulation index, and radius of curvature. Characteristics that follow a Gaussian/Normal Distribution are indicated with a +. For such variables, mean ± SD are presented, and cohort differences are compared using a Student t-test. For variables that do not follow a Gaussian distribution, median and inter-quartile range (IQR) are presented and cohort differences are compared using a Mann-Whitney test.

ACEI indicates angiotensin converting enzyme inhibitors; ARB, angiotensin receptor blocker; BMI, body mass index; CAD, coronary artery disease; CCB, calcium channel blockers; CKD, chronic kidney disease; HDL, high density lipoprotein; HTN, arterial hypertension; Hx, history of; IQR, Interquartile range; LDL, low density lipoprotein; MAP, mean arterial pressure; PAD, peripheral arterial occlusive disease; PAOD, peripheral arterial occlusive disease; RC, radius of curvature; SD, standard deviation; TG, triglyceride; UI, undulation index.

In the second arm, we utilized the clinical database (Oxnet Janus), which prospectively registered every patient who underwent elective repair of AAAs at the John Radcliffe Hospital, Oxford, UK. Each of the patients gave written consent for the utilization of clinical images collected during the routine clinical management pathway for research analysis. Radiology records of all non-emergency infra-renal AAA repairs (open surgery or endovascular repair) from February 1st, 2009 to June 30th, 2018 were examined. Only those patients with at least 1 historic CT scan conducted greater than 8 months before the preoperative scan were included for analyses.

### Automated Segmentation of CT images

AAA segmentation was performed using a proprietary automated deep learning segmentation platform.^18^ The deep learning model generated aortic/aneurysm segmentations which were visually assessed against the source Digital Imaging and Communications in Medicine standard images. Where required, further manual adjustments were performed using the open source ITK-Snap software.^19^

### Extraction of Geometric Features

The geometric features included in our analyses were derived from the aortic/aneurysm wall and can therefore be derived from either contrast or non-contrast enhanced CT images. All geometric features were extracted from the above generated volume segmentations using MATLAB. AAA size was measured by calculating the maximum anteroposterior diameter (APD). Annual aneurysmal growth was derived by subtracting the historic/baseline AAA size from pre-operative AAA size and dividing the difference by the time duration (years) between scans (Annual Growth = ∆ AAA size in mm/(number of days lapsed between scans/365 days)).

### Maximal APD

Maximum APD was automatically extracted from the aneu-rysmal segmentations using MATLAB. For each axial slice along the aortic volume, the diameter was obtained by measuring the maximum distance between 2 points on the aneurysmal boundary in the sagittal plane. The maximum APD of the AAA was the maximum value from all axial slices.

### Radius of Curvature

**Figure 1:**
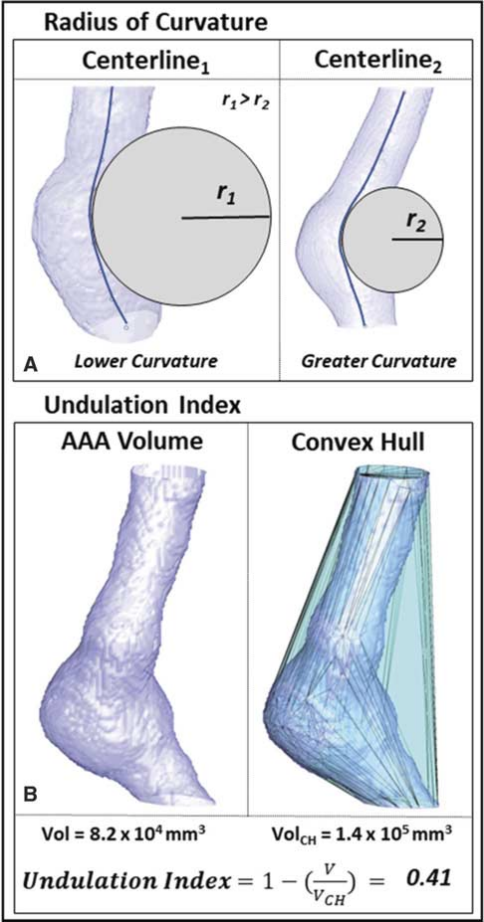
Radius of curvature (RC) (A) and undulation index (UI) (B) measurements obtained from abdominal aortic aneu-rysm (AAA) volumes.

RC is a centerline-based metric that captures the degree of curvature along the centerline.^16^ Here, RC equals the radius of the circular arc that best approximates the curve between a set of adjacent points. The smaller the circular arc, the smaller the RC and the greater the local curvature (Fig. 1A). On the other hand, the larger the circular arc, the greater the RC, and the lower the local curvature (ex. a straight line). AAA centerlines were calculated using an implementation of the homotopic thinning algorithm^20^ and were subsampled using b-spline interpolation methods based on the number of axial slices. RC was calculated for adjacent sets of triplet points and the minimum value was obtained. This described the greatest region of curvature within the aneurysmal volume.

### Undulation Index

Aneurysmal UI was defined as $\text{UI}=1-\left(\frac{V}{V_{CH}}\right)$ .^13,14^ Here, V is the volume of the infra-renal abdominal aorta defined as the region between the renal arteries and the iliac bifurcation and V_CH_ is the volume of its convex hull (Fig. 1B). In this instance, the convex hull of AAA is the smallest volume that encompasses the entire region and is convex at all points. It effectively resembles a plastic wrap attached to the inlet and stretched over the entire aneurysmal surface. This parameter captures the degree of surface concavity and increases with surface/shape irregularity. Conversely, a shape that is nonconcave (ex. a perfect sphere) will have a UI of 0.

### Developing the Growth Prediction Models

To ascertain if APD, UI, and RC were independent of the patient demographic profile for the purpose of AAA growth prediction, we first examined the relationship (Spearman correlation) between these extracted geometric features against demographic features in the prospectively recruited cohort. We then focused the CT image analysis in the second arm of the cohort, using the geometric features extracted from the baseline scan to predict future growth (as recorded by the subsequent preoperative scan).

Summary statistics are described either as average (+/– standard deviations) or median (with interquartile range, IQR). For statistical comparisons, t-tests or ANOVA were used for normally distributed data, whereas Kruskal-Wallis test or Spearman correlation were used as nonparametric tests. Multinomial logistic regression and multiple linear regression models were applied to build the AAA growth prediction models, for the prediction of AAA growth as a categorical (slow, some, fast growth) or continuous variable. Data from the second arm of this cohort was randomly split into training (n = 100) and testing (n = 92) sets. Model training and optimization were performed with the training cohort with 10-fold optimization. Here, the data is partitioned into 10 equal sized subsets (folds). Then, 9-folds are used to train the model and the remaining fold is used for internal evaluation. This process is performed a total of 10 times, with a different fold as the internal validation data set. The optimal parameters were selected, which are then used for the test cohort. Here, the optimized models were used to predict AAA growth on the independent testing cohort (n *=* 92). Model performance was evaluated using correlation coefficient analysis and root-mean-square-error (RMSE) difference between the predicted and measured AAA growth rates.

For the logistic regression model, growth rates were categorized into 3 groups: <2.5 mm/yr (slow growth), 2.5–5.0 mm/yr (some growth), and >5.0 mm/yr (Fast growth). These thresholds were chosen based on the summary statistics of growth rate observed within this cohort. Receiver operating characteristic curves were plotted for each combination on the testing cohort to assess the performance of the regression model in discerning growth against a pre-defined growth rate threshold. Accuracy of the linear regression model was reported using RMSE between the actual annual growth versus predicted growth. We further assessed the prediction accuracy within a 2 mm margin as this is the accepted technical variability between measurements of AP Diameter in CT images.^21,22^

## Results

### Patient Demographic Features Do Not Influence the Geometric Features of AAA

One hundred two patients were included the prospectively recruited arm of this cohort (Male n = 99; Females n = 3). The median age at the time of consent was 72 (IQR: 67–79) years old. The majority were ex-smokers (67%) and 24% were current smokers. A history of symptomatic atherosclerotic arterial disease was prevalent in this group (ischaemic heart disease: 32%; peripheral arterial occlusive disease: 16%; cerebral vascular disease: 12%). The majority of participants reported a prior diagnosis of arterial hypertension (73%) and hypercholesterolemia (60%). However, these were well controlled by long term pharmacological therapy [anti-hypertensive(s): 67%, statin: 73%, anti-platelet(s): 46%], as reflected by their controlled mean arterial pressure (102 ± 13 mm Hg) and overall normal cholesterol profiles [median = 3.8mmol/L (IQR 3.2–4.6), lower than 5.2mmol/L in 82% of participants] at the time of recruitment. Sixteen percent of the participants reported a history of diabetes mellitus, and 27% had chronic kidney disease with eGFR < 60. Baseline demographic data from the prospective cohort (n = 102) are presented in Table 1. Median AAA size within this cohort was 63.0mm with an interquartile range from 58.0 to 72.5 mm. No correlations were identified between the demographic parameters and the extracted geometric features, indicating that AAA size or APD, UI, and RC are independent of patient demographic characteristics.

### Prediction of AAA Growth Using Geometric Features

The second arm of this study included 192 AAA patients with serial CT scans obtained at least 8 months apart. Median follow-up time between scans was 2.0 years with an interquartile range of 1.03.7 years. Similarly, median aneurysmal growth was 3.7 mm/yr with an interquartile range of 2.5 to 5.0 mm/yr. These were randomly split into training (n = 100) and validation (n = 92) sets. There were no differences in the follow up duration and geometric indices between the training and validation datasets (Table S1, http://links. lww.com/SLA/C839).

**Figure 2:**
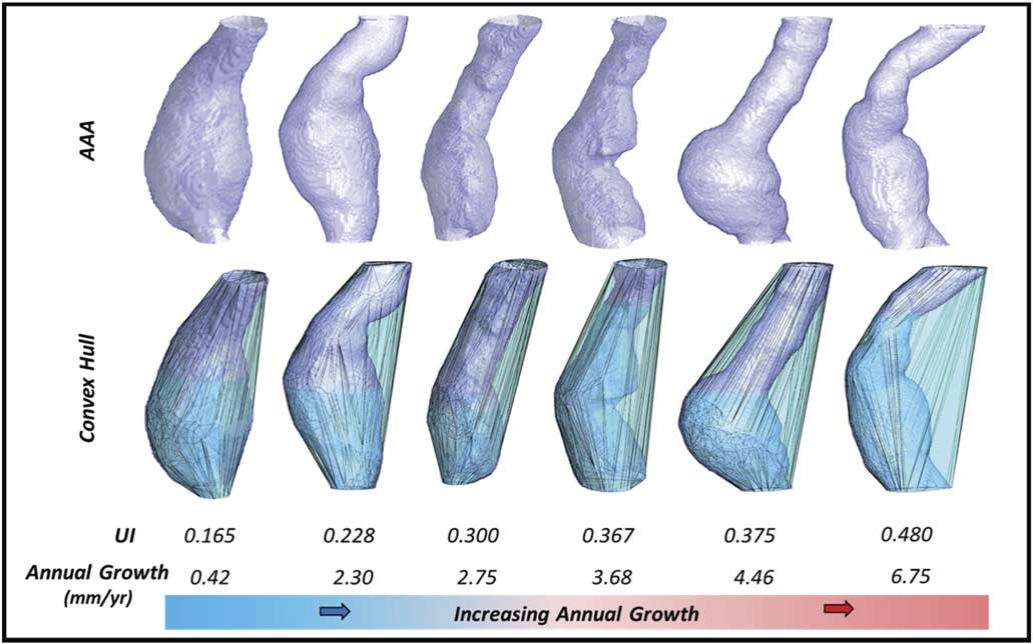
Six AAAs of similar size displayed alongside their respective convex hull. Undulation indices are calculated for each aneurysmal pair. Aneurysms are ordered in terms of increasing UI and positively correlate with increasing annual AAA growth rate. AAA indicates abdominal aortic aneurysm; UI, undulation index.

**Figure 3:**
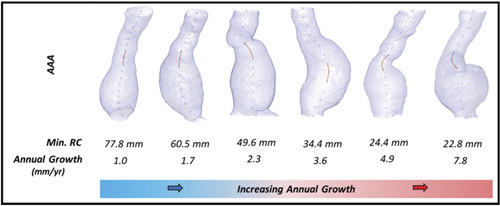
Six AAAs of similar size displayed with calculated centerline. Centerline are calculated using a variation of the homotropic thinning algorithm. Regions with the minimum radius of curvature along each centerline are highlighted in orange. These location correspond to region of increased curvature along that respective centerline. Aneurysms are ordered in terms of decreasing min RC and negatively correlate with increasing annual AAA growth rate. AAA indicates abdominal aortic aneurysm; RC, radius of curvature.

There were significant positive correlations between AAA size (Spearman *r* = 0.25, *P <* 0.05) and UI (Spearman *r* = 0.38, *P <* 0.001) with annual AAA growth rate (Fig S1A and B, http://links. lww.com/SLA/C839). Whereas a significant negative correlation between minimum RC and annual AAA growth rate was observed. (Spearman *r*=-0.53, *P <* 0.001, Fig S1C, http://links.lww.com/ SLA/C839). Figures 2 and 3 illustrates 6 AAAs ofsimilardiameterat baseline with disparate UI and RC. These corresponded to different annual growth rate observed subsequently.

### Prediction of AAA Growth as a Categorical Outcome

The baseline characteristics of the subgroups of patients [(slow growth: <2.5 mm/yr); (some growth: 2.5 mm-5 mm/yr), (fast growth: >5 mm/yr)] are summarized in Table S2, http://links. lww.com/SLA/C839. There was no difference in the starting AAA diameter between the 3 groups. UI at baseline differed significantly between the 3 groups (ANOVA, *P* = 0.003). There was also a significant inverse trend of relationship between RC and AAA growth (Kruskal-Willis, *P* = 0.003).

**Figure 4:**
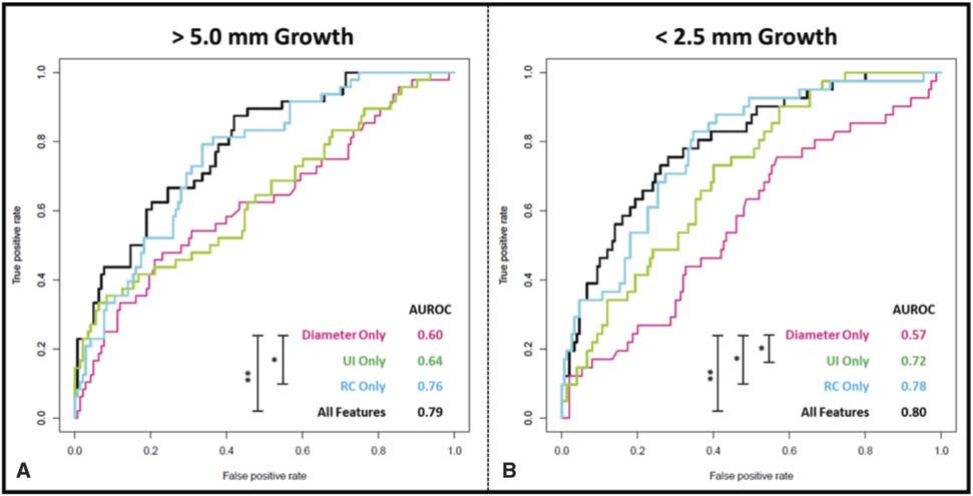
Receiver operator characteristic (ROC) curves for each combination of features alongside the area under curve (AUROC) to assess the performance of the multinomial regression model in discerning AAA growth phenotype (A, >5.0 mm and B, <2.5 mm Growth). ROC curves were generated from the evaluation of the test cohort (n = 92), following model training. Significance testing was performed to compare individual ROC curves and significant differences were noted with * or **indicating *P <* 0.05 and *P <* 0.01, respectively. AAA indicates abdominal aortic aneurysm.

Different combinations of input features (APD, UI, and RC) were used to train multiple logistic regression models. The feature combinations used for each model is indicated in Figure 4 and were trained using a 10-fold cross-validation approach. Receiveroperating characteristic curves on the testing cohort were plotted for each feature combination with the threshold of “Slow (<2.5 mm) Growth” and “ Fast (>5.0mm) Growth” (Fig. 4). The area under receiver operation curve metric shows good discriminative capacity of AAA growth rate based on all 3 variables at the predefined thresholds. Using APD, UI, and RC as 3 input variables of the prediction algorithm, the area under receiver operation curve for predicting slow growth (<2.5 mm/yr) and prediction fast growth (>5mm/yr) is 0.80 and 0.79, respectively. This model comprising of 3 variables significantly outperforms the use of AAA diameter alone as the predictor *(P* < 0.01).

### Prediction of AAA Growth Rate as a Continuous Variable

**Figure 5:**
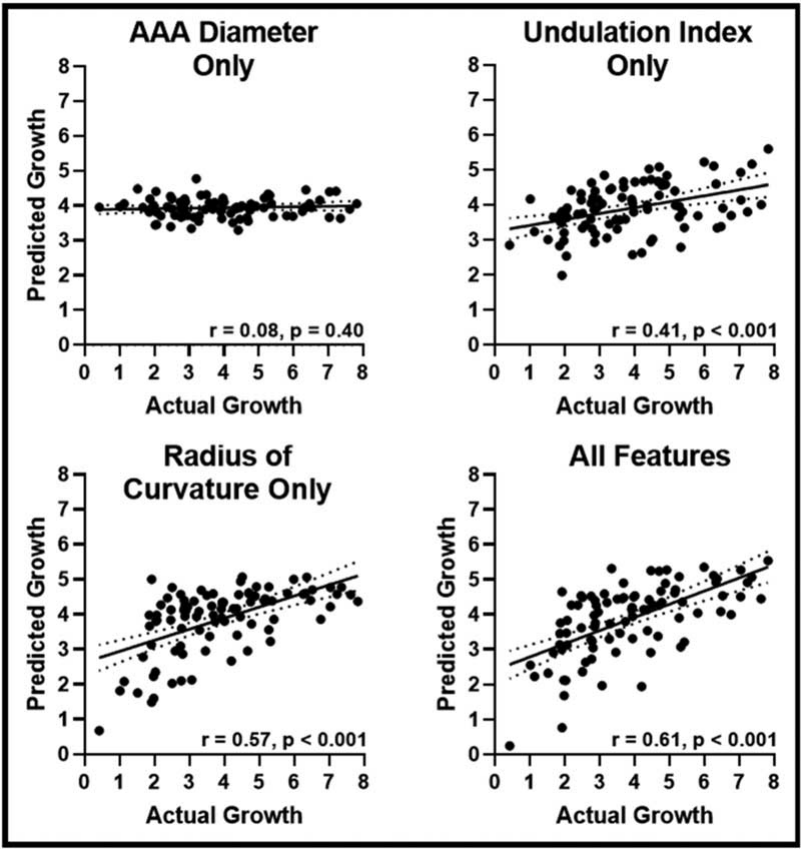
Outputs of linear regression models trained to predict annual AAA growth. Four Linear regression models were trained using 10-fold cross-validation to predict annual AAA growth. Models were evaluated using the testing cohort (n = 92) and are reported against actual AAA growth values. Statistical significance is assessed for each correlation and is indicated on each graph. Dotted lines indicate the 95% confidence interval for the regressions. AAA indicates abdominal aortic aneurysm.

Linear regression models were trained and optimized simultaneously using a 10–fold cross-validation approach to predict AAA growth rate (mm/yr) as a continuous variable. Similar to the multinomial logistic model, the linear model trained using Max AP diameter, UI, and RC was able to predict annual AAA growth to a greater accuracy than the model trained using only max AP Diameter (Fig. 5). Predictions from this model were significantly correlated (r = 0.61, *P <* 0.001) and closer (RMSE: 1.32 ± 1.44 mm) to that of observed measurements than that of the other models. Model performances on both train and test cohorts are summarized in Table 2. Similarly, this model is able to predict annual AAA growth to within 2 mm error in 87% of cases.

**Table 2:** Evaluation of Linear Regression Models to Predict AAA Growth

	Train (n = 100)			Test (n = 92)		
Features	RMSE	Correlation	*P*	RMSE	Correlation	*P*
AAA Diam Only	1.52 ± 1.70 mm	0.25	0.03	1.72 ± 1.85 mm	0.08	0.40
Undulation Index Only	1.44 ± 1.66 mm	0.38	<0.001	1.53 ± 1.68 mm	0.41	<0.001
Radius of Curvature Only	1.32 ± 1.50 mm	0.52	<0.001	1.38 ± 1.68 mm	0.57	<0.001
AAA Size + UI + Min. RC	1.26 ± 1.50 mm	0.59	<0.001	1.32 ± 1.44 mm	0.61	<0.001

Four linear regression models were trained using 10-fold cross-validation to predict annual AAA growth. Models were evaluated using the validation folds of the training cohort (n = 100) and testing cohort (n = 92). Model Predictions of AAA growth were compared against observed values using root-mean-square error and correlation coefficient analysis. Significance of correlations are reported.

AAA indicates abdominal aortic aneurysm; RC, radius of curvature; UI, undulation index.

## Discussion

In the United Kingdom, patients diagnosed with an AAA through the National AAA screening program are subject to as frequent as 3-monthly surveillance scans.^23^ Surveillance of screening detected or incidentally-diagnosed AAA is also standard clinical practice as recommended by international guidelines.^1,2^ Furthermore, in aging populations within developed countries, the health burden of AAA surveillance is expected to rise. As an example, the National AAA screening program alone incurs a net increase of ~2000 patients requiring AAA surveillance each year.

Methods to predict future aneurysmal growth are valuable to both clinicians and patients. Such tools can improve the stratification of AAA surveillance frequency in individuals: those with slow growth AAAs should not require as frequent surveillance, whereas intense surveillance (or early intervention) can be justified with an AAA that is likely to exhibit rapid growth. Here, we present a method of AAA growth prediction which utilizes geometric features derived from clinical CT scans.

Recent reports have investigated the use of biomechanical assessments of aortic aneurysms from medical images to predict rupture risk potential. Doyle et al. investigated the use of a ratio between aneurysm wall stress to wall strength, derived from magnetic resonance images images, to estimate rupture risk.^24^ Their results supported an increase in AAA-related intervention in cases with an elevated ratio after adjusting for AAA diameter and other clinical factors. Additionally, Martufi et al, studied the impact of AAA features (ex. lumen/vessel volume, intraluminal thrombus thickness, etc) and derived wall stress measurement on AAA outcome prediction.^25^ Their results display slight but significant improvements to contemporary methods.

However, such biomechanical models require specific assumptions of physiological/computational variables for each individual patient, which has inherent variability but also may not hold true in real life context.^26,27^ For example, the aneurysm biomechani-cal ratio calculated by Doyle et al is subject to many levels of uncertainty including variability with (1) image reconstruction accuracy, (2) principal wall stress estimation, and (3) population-based wall strength estimation. This results in significant overlap between results for asymptomatic and symptomatic/ruptured cases and prevents clinical implementation.^28^ Therefore, it is difficult to draw conclusions about this ratio and other similar wall-stress methods for patient-specific prediction of AAA events. This is further supported by Leemans et al., which suggests that biomechanical indices present no added value in the AAA rupture risk assessment.^29^ Wall-stress evaluation was not a component of this study and all geometric features were derived directly from the aneurysmal volume.

There is no prior literature on the prediction of AAA growth using complex geometric features extracted from CTs. This has been examined in the context of cerebral aneurysms. Dhar et al, investigated the use of image-based morphological features to predict intracranial aneurysm rupture.^13^ Their study highlighted the importance of 6 features that capture the morphological diversity of intracranial aneurysms and their relation to inflow/outflow vessels. Since then, many of these features have been shown to alter underlying hemodynamics and promote intracranial aneurysmal rup- ture.^13,14,30^ Although there are many similarities between intracranial and aortic aneurysms, these features have not been investigated in the latter. One such parameter, UI captures the degree of surface concavity and increases with surface irregularity.^13,14^ A highly asymmetric and/or tortuous infra-renal region would result in an increased UI parameter.

In this study, high undulation seems to arise from a “bent” (correlated with curvature), “bulgy” (a thin cylinder directly attached to a sphere, like the fifth case in Fig. 3) and/or“undulated” shape (2 or more spheres attached by cylinders). This parameter is specific to the aneurysmal infra-renal region of the aorta and does not require additional information including inflow/outflow volumes. Similarly, minimum RC is a descriptor of the AAA centerline, which captures the region of maximum curvature. Here, RC is radius of the circular arc that best approximates the curve between a set of adjacent points. The smaller the circular arc, the smaller the RC and the greater degree of local curvature.

The advantage of the geometric measurements described here (APD, UI, RC) is that they can be readily extracted from either contrast or non-contrast CT images, without specific adjustment in CT scanning protocols. This analysis is streamlined by our proprietary automated pipeline for high resolution segmentation of blood vessels using deep learning approaches.^18^ That no correlation was observed between these geometric features to the patient characteristics (as summarized in Table 1) further supports our claim that they can be independently deployed as predictive indices without accounting for patients' demographic characteristics. Further validation of our prediction algorithm can therefore be attempted using historic scans already accumulated by vascular surgical units who have an existing clinical image database as part of their routine AAA management practice.

There is emerging literature on the role of best medical therapy (BMT) in reducing overall mortality risk in patients with a AAA, exemplified by the VIVA study long term data.^31^ Our method of AAA growth prediction can complement the delivery of BMT in the surveillance pathway. Those predicted to have fast growth should warrant further targeted intensive BMT regime to change their risk profile. Of note, in the prospectively recruited arm of this study, only a small fraction of patients included were females. We therefore could not rule out an association between these geometric features to the reported demographic features in female patients.

Although AAA surveillance is typically performed using ultrasound scans, many of these patients undergo CT scans for other clinical reasons during the course of their AAA surveillance. These CT scans can be utilized for the added purpose for the prediction AAA growth. (Of note, AAA surveillance is indeed performed using serial CT scans in countries such as Japan.) With the refinement of CT imaging technology and reduction of radiation dose per scan and portable tomographic CTs, it is not implausible for CT scans to replace ultrasound scans as the choice of AAA surveillance in the future. This will facilitate the development and validation of CT image derived prediction algorithms.

## Conclusions

We present an AAA prediction model which utilizes geometric features that can be readily extracted from clinically acquired CT scans. This method can be applied to historic scans acquired during the routine clinical pathways of each AAA patient.
